# Experiences of quality of life and access to health services among rare disease caregivers: a scoping review

**DOI:** 10.1186/s13023-024-03327-2

**Published:** 2024-08-31

**Authors:** Tina Černe, Lijana Zaletel Kragelj, Eva Turk, Danica Rotar Pavlič

**Affiliations:** 1 Department of Family Medicine, Medical Faculty, University of Ljubljana, Poljanski nasip 58, Ljubljana, 1000 Slovenia; 2 Department of Public Health, Medical Faculty, University of Ljubljana, Zaloška cesta 4, Ljubljana, 1000 Slovenia; 3 Center for Digital Health and Social Innovation, University of Applied Science St. Pölten, Campus-Platz 1, St. Pölten, 3100 Austria; 4 Medical Faculty, University of Maribor, Taborska 8, Maribor, 2000 Slovenia

**Keywords:** Rare diseases, Caregivers, Quality of life, Access to health care

## Abstract

**Background:**

Research on rare diseases focuses less on caregivers, who play an important role in meeting the medical and social needs of the people they care for. Caregivers of people with rare diseases face negative outcomes due to problems with diagnosis, caring for complex conditions and expensive treatments. However, the factors that affect their quality of life are poorly understood. Poor mental and physical health of caregivers has a direct impact on the person they are caring for.

**Methods:**

To explore the literature on this topic, we conducted a scoping review in which we identified and analysed relevant studies to find out how extensively this topic has been researched. The articles were retrieved from the bibliographic databases PubMed, Ovid Medline and Ebsco Cinahl.

**Results:**

We initially identified 299 references and then included thirty-four articles. The included articles address three main topics, namely caregiver quality of life, health care accessibility, and the impact of health care accessibility on caregiver QOL.

**Conclusion:**

This study provides information that is important to multiple providers of services as it can help to better understand caregivers and people with rare diseases and improve the quality of services offered. It highlights areas with the greatest need for change and offers insight into the complexity of caring for people with rare diseases, assisting policymakers in developing policies to support informal caregivers.

**Supplementary Information:**

The online version contains supplementary material available at 10.1186/s13023-024-03327-2.

## Background

In Europe, rare or orphan diseases are defined as diseases with a prevalence of less than 5 per 10,000 people in the population [66]. The estimated number of different rare diseases is currently around 10,000 [32]. While the incidence of individual diseases is low, the collective prevalence of all types of rare diseases is high [76]. The Council of Ministers of the European Union estimates that 6 to 8% of the European population will be affected by a rare disease in their lifetime [58]. For most rare diseases, appropriate medical interventions have not yet been developed or the treatment is still unknown [79]. Although EU rare disease policy has been successful, rare disease stakeholders agree that there are still significant problems with access to orphan drugs at national level and that there are major inequalities in this area [61].

People with rare diseases and their families face the challenges of delayed diagnosis, difficult access to health care, and financially unmanageable treatment [74]. With the exception of studies focusing on specific diseases and their pathophysiology, there are few studies looking at the experiences of rare disease caregivers [16]. Caregivers of people with rare diseases are usually parents or spouses. They bear most of the physical and emotional burden of caring for a person and usually receive no financial compensation for their role [16]. In addition, many are forced give up or reduce their jobs because they have to take on the responsibility of caregiving, which can lead to additional financial problems [8, 46].

Caregiving can involve many adjustments in a caregiver’s life, such as providing transport, running errands, providing emotional support, monitoring symptoms, taking on additional household tasks and adapting to a special diet [10, 73]. Caregivers play an important role in the daily management of the disease. Their physical and mental state has a direct impact on the level of care they can provide to the person with the rare disease. Providing informal care can lead to unimaginable emotional, social and physical health outcomes [8, 51, 53, 64, 76].

Recently, more attention has been paid to caregivers of people with rare diseases and their quality of life (QOL). It has been shown that the role of caregivers exposes them to many negative effects resulting from the problems of diagnosis, care of complex diseases and the difficult and expensive treatment regime [8, 25, 51].

Studies investigating the QOL of caregivers have mostly conducted for specific rare diseases. Therefore, there is a lack of a synthesis of results that examines the factors that influence the QOL of caregivers. Knowledge about rare diseases is low, not only among the general population but also among healthcare providers [69]. Few studies have been conducted to determine how access to healthcare services affect caregivers of people with rare diseases. It has been shown, that people with rare diseases, regardless of their disease, face the same problems in accessing healthcare [43]. They encounter barriers to accessing appropriate information and specialists (who may be located in other countries) [76].

In our daily practice interacting with caregivers and people with rare diseases, we have found that there is a great need for better support and understanding of these issues and a lack of knowledge about the challenges of caring for people with rare diseases. It is therefore crucial to identify clear areas for change that should be evidence-based to help researchers, healthcare providers and other service providers develop, plan and deliver better services. To gain more information about how barriers to accessing health care affect caregivers’ QOL and to identify key areas for change. We conducted this study to learn more about how barriers to accessing healthcare affect caregivers’ QOL and to identify key areas for change. This study is one of the few to examine the relationship between QOL and barriers to access to healthcare for caregivers with rare diseases, filling a gap in international research. By exploring these questions, we aim to contribute to a broader understanding of caregiving around the world and provide valuable insights for policy makers, healthcare providers and researchers worldwide.

The aim of this study was to conduct a comprehensive literature review to address two key research questions: (1) the impact of access to health care services on the quality of life (QOL) of informal caregivers of individuals with rare diseases, and (2) the factors associated with QOL and access to health care services among informal caregivers of rare diseases.

## Methods

Due to the exploratory nature of our research questions, we used a scoping review method for this study. As described by Arksey and O’Malley [6], scoping reviews serve to identify and map the available evidence on a particular topic in order to obtain a comprehensive overview of the literature. Our aim is to explore the relationship between quality of life and access to healthcare for caregivers of people living with rare diseases, and the scoping review approach fits with our aim to capture the broad landscape of literature on this topic [6, 57]. With this review, we aim to highlight key concepts, features and knowledge gaps in the existing literature to providing a foundation for future research [6, 57]. The methodological framework for scoping reviews proposed by Arksey and O’Malley guided us through five key steps [7].

### Identification of the research question

In this study, we answered the following two research questions: (1) How does caring for a person with a rare disease affect the caregivers QOL? (2) How does access to healthcare services affect the QOL of caregivers of people with rare diseases?

### Identification of relevant studies

For the literature search, journal articles published between 2005 and 2021 were searched using keywords in the following electronic databases: PubMed Central, PubMed, Ovid Medline, and Ebsco Cinahl. This time frame was chosen in order to capture the latest developments while ensuring comprehensive coverage of the existing literature. Literature searches were conducted in November and December 2021. We searched the database PROSPERO to ensure that no similar studies had been started or were planned. Search term strategies can be found in the additional files (refer to Additional file 3).

### Study selection

A reviewer reviewed and selected the titles and abstracts that emerged from the database searches. The full texts of the selected references were then retrieved, reviewed, and article selection followed. In addition to the search strategy, we manually searched the reference lists of the included articles. To ensure proper selection of articles, some of the full-text publications were subsequently reviewed by another independent reviewer. For access to other primary sources and full-text versions of articles, we used Google Scholar and ResearchGate. The complete list of inclusion and exclusion criteria with descriptions can be found in Additional file 4.

### Charting the data

A data extraction sheet was developed to record information on authors, year, country and to identify the main themes in the included articles. We recorded the information as follows: (a) Study characteristics: authors, year and country of publication, (b) study objective, (c) study design, study methods, type of QOL instruments, (d) sample size, age and sex distribution, (e) type of rare disease, (f) common themes, (g) study results. We also reported the type of sample if the study design was qualitative. Summary and evidence tables were created for this purpose. To determine whether the diseases in the included articles were rare, we checked whether the prevalence of the disease met the European definition of rare diseases, which is defined by a prevalence of less than 5 per 10 000 people in the population [66].

### Collating, summarizing, and reporting the results

In the fifth and final phase of the review, information was collected on caregivers’ QOL, access to healthcare and their possible relationship. As the studies were too heterogeneous, we undertook a narrative synthesis of the results.

## Results

### General description

**Fig. 1:**
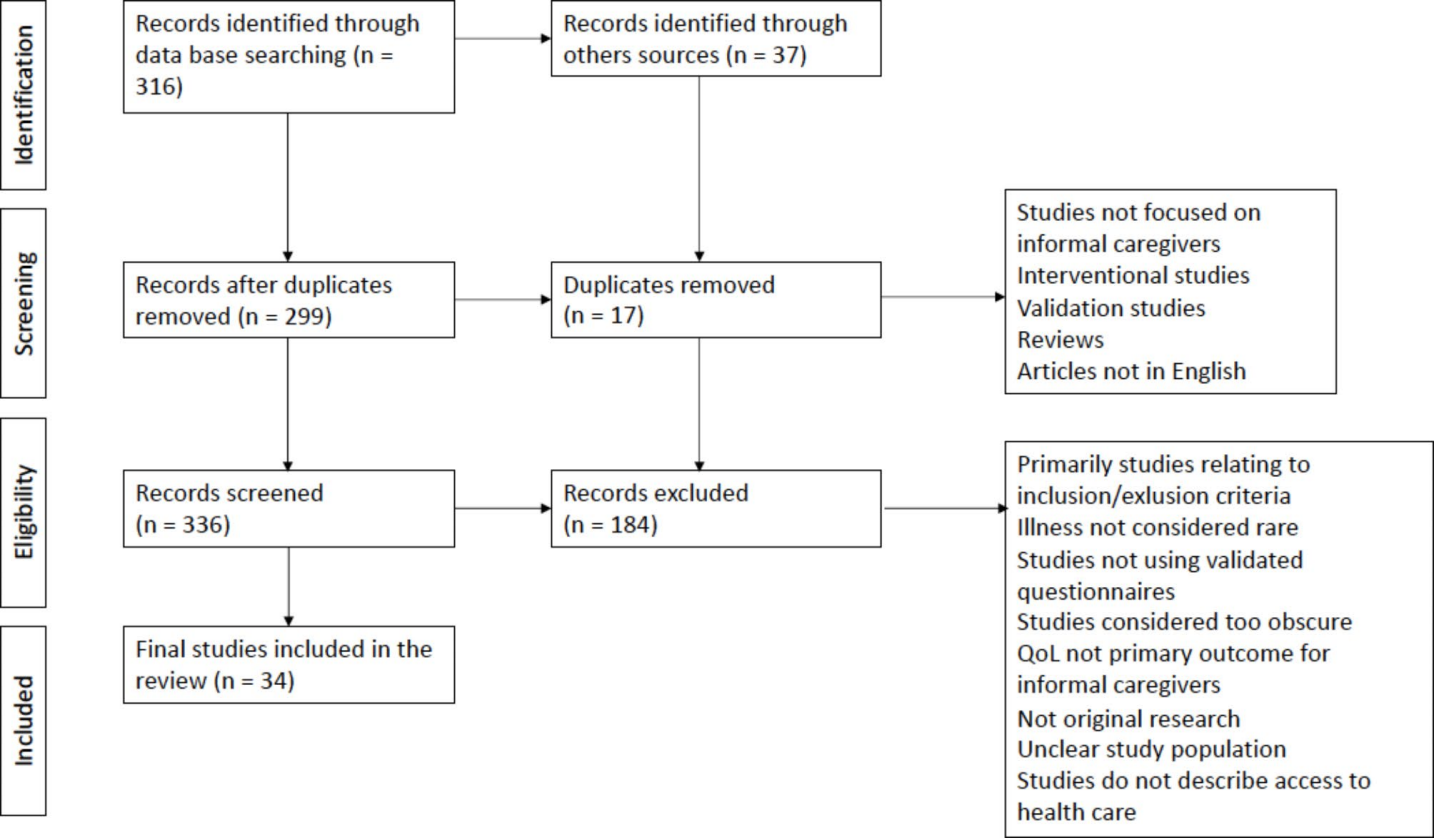
Flow diagram of inclusion and exclusion process in the scoping review

During the literature search, we identified 299 articles after removing duplicates. 183 articles were excluded based on title, and another 119 after reviewing the full text. The search was carried out from 22 November to 23 December. Finally, after reviewing the content of the remaining articles, 34 articles matched the searched topic. The procedure for selecting articles for a scoping review is show in Fig. 1.

### Study characteristics

A total of 34 studies met the inclusion criteria, which are listed in Table 2: Details of selected articles found in the literature search on the QOL of rare disease caregivers; and Table 3: Details of selected articles on access to health services for rare disease caregivers (see Additional file 1 and Additional file 2). Twenty-four studies used quantitative methods, 6 studies used qualitative methods and four studies used a mixed methods approach. The quantitative studies primarily used questionnaires and, in some cases, online surveys (*n* = 24) to collect data; the qualitative studies used face-to-face interviews (*n* = 5) and focus groups (*n* = 1) to collect data. The mixed methods studies used a combination of interviews (focus groups, face-to-face interviews, telephone) and questionnaires and online surveys.

**Fig. 2:**
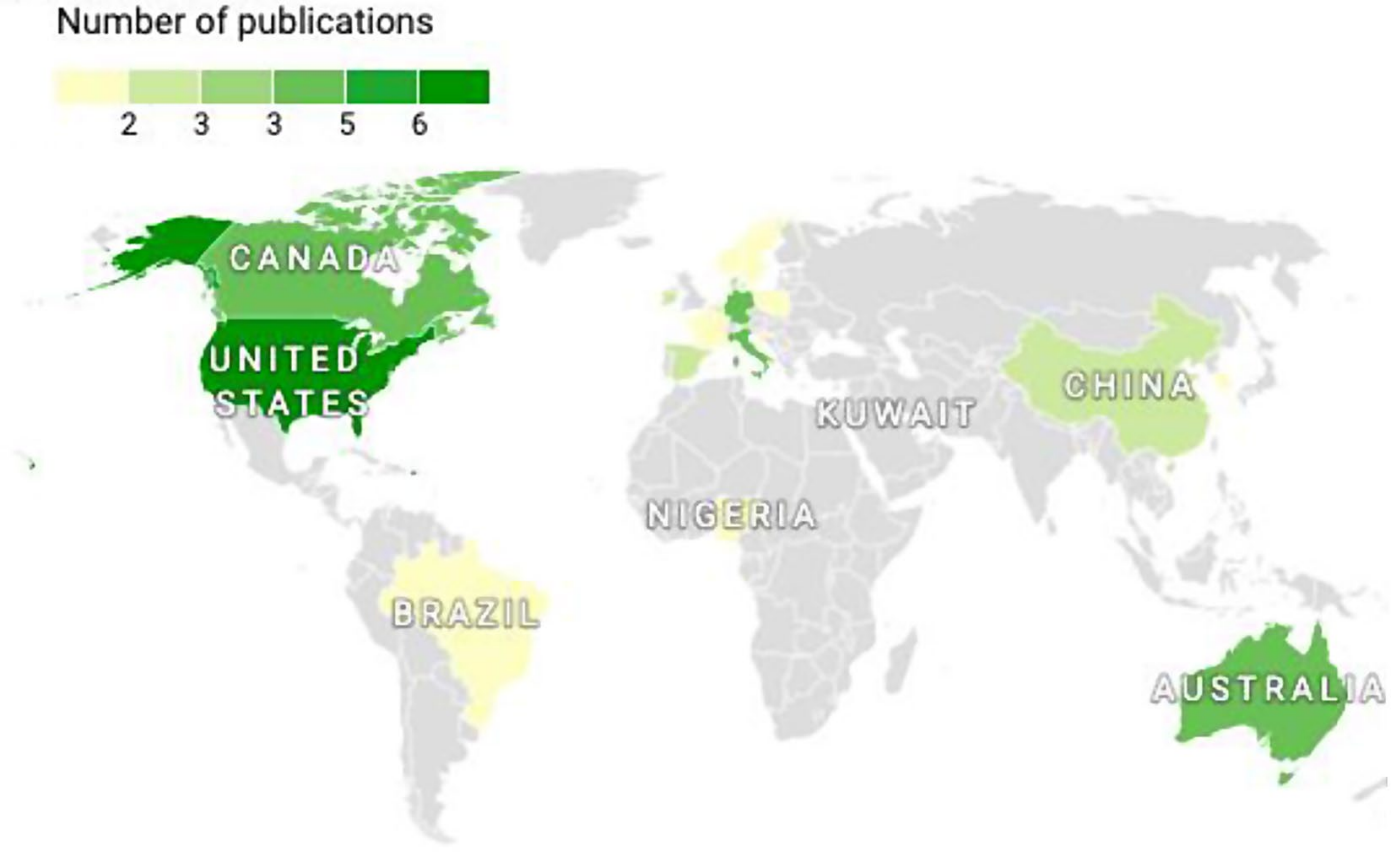
Country origin of studies included in the scoping review

**Fig. 3:**
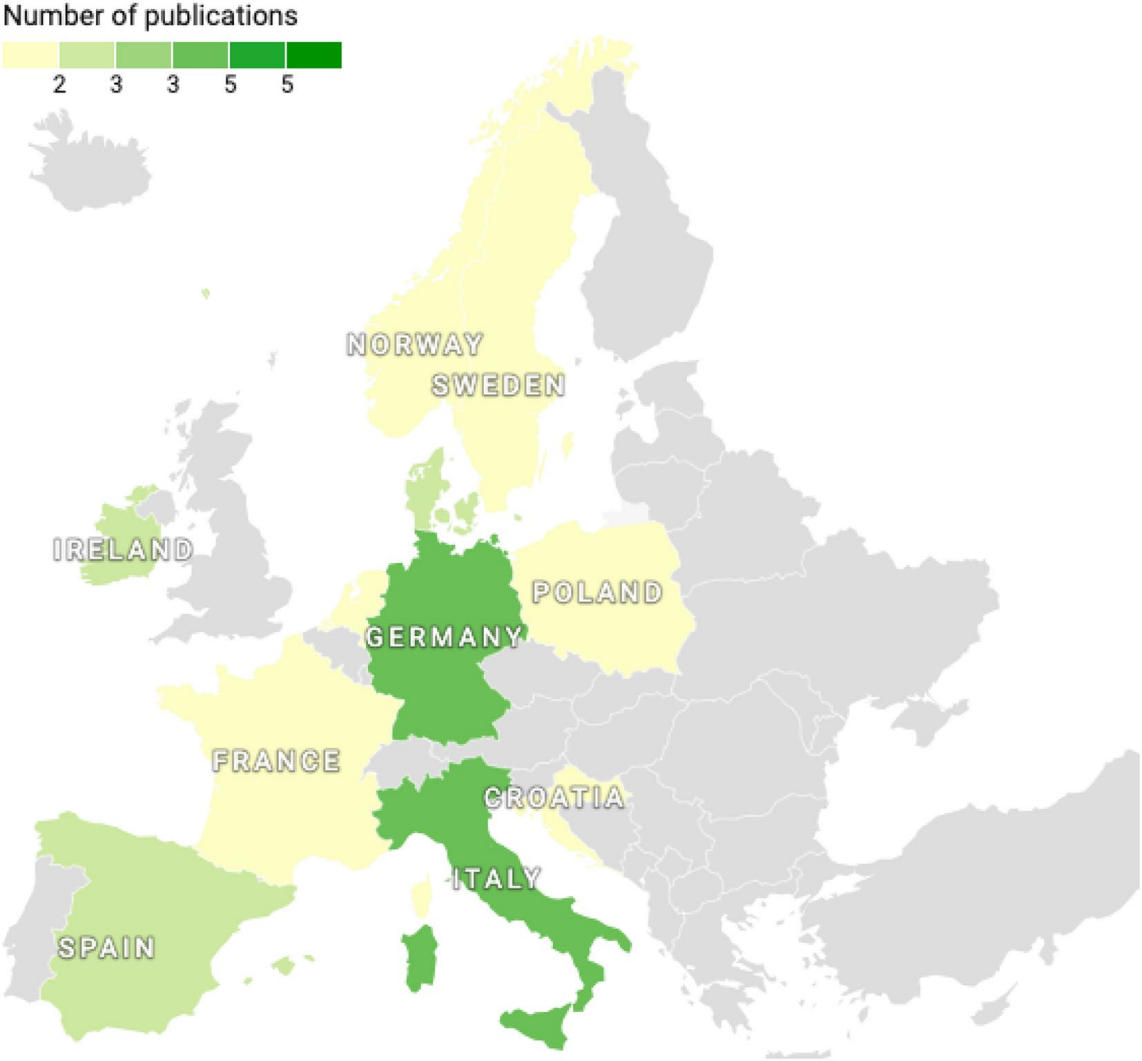
European countries included in the review

The included studies were from 18 different countries. Figures 2 and 3, showing the choropleth maps, clearly illustrate the samples included in this review. A detailed description of the included studies and the countries of origin can be found in the Additional file 5 (see Additional file 5).

Fifteen different questionnaires were used to analyse QOL in the studies and are listed in Table 1 The gender distribution showed a higher proportion of women compared to men in most studies. Sample sizes in the included quantitative studies varied widely, ranging from 12 to 952 caregivers and from 5 to 33 caregivers in qualitative studies.

**Table 1:** QOL instruments used in studies included in the scoping review

Name	Abbreviation	Number of studies
The Ulm Quality of Life Inventory for Parents	ULQIE	1
World Health Organization Quality of Life Bref	WHOQOL-BREF	7
WHOQOL Spirituality, Religiousness, and Personal Beliefs	WHOQOL-SRPB	1
EQ-5D-5 L	EQ-5D-5 L	2
The Beach Center Family Quality of Life Scale	FQOL	1
McGill Quality of Life questionnaire	MqoL	4
36-Item Short Form Survey	SF-36	2
12-Item Short Form Survey	SF-12	2
Care Related Quality of Life	CarerQol	2
EQ-5D-3 L	EQ-5D-3 L	1
Short-Form-8 questionnaire	SF-8	2
The Schedule for the Evaluation of Individual Quality of Life-Direct Weighting	SEIQoL-DW	1
CareGiver Oncology Quality of Life questionnaire	CarGOQoL	1
Assessment of Quality of Life	AQoL	1

### Results addressing QOL

We discovered three themes related to caregivers’ QOL. The first was the QOL of caregivers compared to other groups, such as caregivers of chronically ill or healthy control experts. The second was the dimensions of QOL that were most affected, and the third was the factors that influence the impairment of QOL.

#### QOL of informal caregivers in compared to other groups

Eight studies reported that the QOL of caregivers decreased significantly [1, 5, 28, 37, 49, 54, 67, 82], seven studies also found that the QOL of caregivers was significantly lower than that of healthy controls [2, 12, 45, 54, 75, 82, 84]. A study by Berrocoso et al. also found that the QOL of informal caregivers of rare diseases was also lower compared to a group of caregivers of chronic diseases [12]. When comparing different types of rare diseases studies by Guarany et al. and Qi et al. found that the QOL of caregivers was significantly lower for more severe forms of rare diseases [31, 65]. In terms of QOL and mental health, four studies reported that mothers appeared to be more affected than fathers [13, 31, 41, 84]. In contrast to the previously mentioned studies, a French study found that the QOL of caregivers was consistently high, i.e. not negatively affected by caregiving [54].

#### Affected dimensions of QOL

In thirteen studies, caring for people with rare diseases led to lower scores in the psychological dimension of QOL, i.e. more anxiety and depression [5, 12, 24, 28, 36, 40, 45, 49, 52, 54, 70, 82, 84]. Informal caregivers often reported that the physical activity dimension was also severely impaired [12, 13, 28, 31, 36, 52, 54, 67, 82]. Four studies also reported that caregiving impacts social QOL, as caregiving burden reduces their engagement in social relationships and with their partners, which in turn negatively affects QOL [21, 40, 52, 70].

#### Factors that influence QOL of caregivers

The QOL of the cared-for person and the informal caregiver appears to be linked. Xu et al. found that the severity of illness perceived by patients is an important factor influencing the health-related QOL of family caregivers [84]. In addition, three studies showed that a positive relationship between the QOL of the caregiver and the QOL of the cared-for person was also a factor [2, 82, 83]. The age of the person being cared for appears to influence QOL of caregivers. A study by Antoniadi et al. found that relative to the patient’s age of at onset of illness, a later onset was associated with a lower QOL score for the caregiver [5]. In addition, studies by Roach et al. and Boettcher et al. found that the age of the person with a rare disease is an important determinant of QOL, with QOL increasing with age, i.e., younger age is associated with poorer QOL [13, 67]. The duration of caregiving was also related to QOL in two studies: the longer the caregiver provided care, the lower the caregiver’s QOL [2, 28], Kanters et al. found this was also true for the number of hours spent providing care [36]. The employment of caregivers affected QOL of caregivers. Rodríguez Bermejo et al. and Alshubaili et al. showed that all those who were unable to do paid work scored higher on measures of psychological distress [2, 68]. Rodríguez Bermejo et al. also found that people who were separated or single had higher scores for feeling overwhelmed than the rest, and single people had lower QOL scores [68], suggesting that marital status has an impact on QOL. Caregiver sleep quality was an important factor of perceived QOL as found by Feeley et al. [24]. QOL of caregivers was also negatively affected when the person they cared for had the same residence [81]. We found two factors that positively influence the QOL of caregivers. In a Chinese study, shared caregiving was found to have a positive effect on improving caregivers’ health-related QOL [84], and three studies found that better education led to better QOL [2, 12, 84].

### Results addressing access to healthcare services

A summary of the results of the individual studies can be found in Table 3. Six studies were qualitative [11, 18, 19, 22, 30, 39], and one was quantitative [33]. Four studies were qualitative phenomenological studies [11, 18, 22, 30], two were qualitative grounded theory studies [19, 39] and one was a cross-sectional study [33].

#### Diagnostic odyssey

Five studies reported that caregivers had difficulty seeking or obtaining a diagnosis [11, 18, 19, 33, 39], and two studies reported misdiagnosis [11, 39]. Hiremath et al. found that caregivers reported medical challenges, such as switching to multiple providers before receiving a diagnosis [33]. In addition, four studies found difficulties accessing various healthcare services (e.g., referrals to specialists, physiotherapy…) [11, 22, 30, 39]. Grut et al. found that the lack of involvement of many different service providers was described by caregivers as particularly difficult when it came to healthcare providers [30]. Due to the lack of treatment options, caregivers felt they had to take whatever they could get, even if the treatment was not licenced, as reported in a study by Kesselheim et al. [39].

#### Lack of knowledge

A common barrier to accessing healthcare services in five studies was a lack of knowledge and information on the part of healthcare providers [11, 19, 22, 30, 39]. In addition, caregivers in six studies reported difficulties in accessing information about the disease and its treatment, either on websites or from healthcare providers [11, 19, 22, 30, 33, 39]. Currie et al. and Grut et al. found that many healthcare providers were encountering this rare disease for the first time [22, 30]. Currie et al. and Grut et al. also found that healthcare providers sometimes made decisions based on their personal assumptions about the disease or were reluctant to refer to the information offered by caregivers and they tended not make the effort to seek additional relevant information about the diagnosis [22, 30].

#### Limited collaboration and integration of health services

To overcome barriers to healthcare, caregivers had to adapt to the role of care coordinator, as coordination between different clinics and specialists was poor, as found in three studies [11, 19, 22]. Caregivers also took on the role of advocate because healthcare providers lacked knowledge and caregivers often knew more than healthcare providers, as reported in four studies [11, 22, 30, 39]. Contact with other caregivers or participation in support groups proved to be a source of information for accessing healthcare services, as noted in three studies [11, 19, 39].

#### Financial issues

Baumbusch et al. and Hiremath et al. found that problems accessing healthcare services affect families’ financial resources as they have to pay out of pocket [11, 33]. A study by Kesselheim et al. also found that another barrier to healthcare in certain countries is the lack of insurance coverage due to insurance companies’ lack of knowledge about the rare disease [39].

## Discussion

Although rare disease research is attracting increasing attention, access to healthcare services for caregivers of people with rare diseases has not been extensively studied. The aim of this review was to examine how challenges in accessing health care relate to caregivers’ QOL. To our knowledge, this study is one of the few studies to examine how QOL is viewed through the lens of barriers to accessing healthcare, with a focus on caregivers.

Our first objective was to investigate how caring for a person with a rare disease affects the caregiver’s QOL. Similar to the study by Boettcher et al. on the QOL of parents of children with rare diseases, we found that caregivers had a poorer QOL than healthy controls and caregivers of people with other chronic diseases [14]. The dimensions most affected by caregiving were psychological, physical and social. Caregivers of people with rare diseases were more likely to suffer from depression and anxiety. Our findings are consistent with a review by Pelentsov et al. which found that caregivers of people with rare diseases often feel physically exhausted suffer from sleep disturbances, fatigue, loss of appetite, weight loss, headaches and frequent colds [62]. Due to caregiving, they experience social isolation, loneliness, and dissatisfaction. Participation in social activities may be further limited by the complexity of the person’s condition and their dependence on medical devices [42]. They often feel that their social life is being cut short, that they are losing their freedom and they yearn for more spontaneity [62]. This points to the problem of the lack of respite services for rare diseases, which would allow caregivers to look after themselves and give them a much-needed break.

We found several factors that influence the QOL of caregivers of people with rare diseases. Consistent with previous research by Boettcher et al. and Pelentsov et al., we found that disease severity, patient age, education, gender, and unemployment are important factors influencing QOL [14, 62].

In addition, we identified several new factors that influence the QOL. Sleep quality was found to correlate with QOL. As already stated by Azizi et al., better sleep quality leads to better mental and physical health and vice versa [9]. The patients perceived severity of illness was also a factor influencing caregivers’ QOL. Patients with more severe symptoms needed more support from their caregivers. It is to be expected that the QOL scores reported by caregivers caring for patients with severe illnesses would be lower than those reported by caregivers caring for patients with milder symptoms [20].

The QOL of caregivers was negatively affected if the person they cared for lived in the same house. This could mean that the person being cared for has a more severe form of the disease and needs more care, which may lead to a greater burden on the caregiver and negatively affect their QOL. These findings are consistent with those of Hughes et al. who found that having a family relationship with the care recipient and living with the care recipient were associated with higher levels of objective burden [34].

Furthermore, this could explain a positive relationship between the QOL of the caregiver and the QOL of the person being cared for. Regarding the age of the patient at the disease onset, later disease onset was associated with a lower QOL score for the caregiver. We hypothesise that this result is related to the fact that the rare disease was diagnosed later in life. This finding is similar to that of Lingen et al. who showed that the final diagnosis improves the QOL of parents whose children have a disability [47]. If this is the case, it could also be that caregivers have not received the necessary information, which is an important factor in predicting caregivers’ QOL.

The finding that a later onset of the diseases is associated with a lower QOL can also be explained by Kenny et al.‘s study, which emphasizes the significant impact on the psychological well-being of caregivers and supports the idea that early diagnosis and psychological support are crucial for better adaptation [38].

Increasing duration of care had a negative effect on QOL, both true for daily hourly care and years of care. As Vitaliano et al. emphasise, the somatic condition of caregivers deteriorates with increasing duration of care and makes them more vulnerable to the negative effects of stress [77]. Marital status was also found to have an impact on QOL. Those who were separated or were single had higher scores for feeling overwhelmed than the rest, and single people had a lower QOL, possibly suggesting that single caregivers experience less social and caregiving support and therefore experience greater strain. This could also explain the finding that shared caregiving had a positive effect on improving QOL. These interpretations would be consistent with previous findings that spouses report the lowest burden of caregiving, suggesting that sole caregiving leads to a higher perceived burden [34].

We have found that mothers have a lower QOL compared to fathers, especially in the psychosocial aspects of QOL. In families with disabled children, the traditional division of roles seems to be more pronounced, meaning that the mother takes on the role of caregiver, which is associated with lower well-being. In addition, Gray et al. have shown that illness in the family can have different meanings for men and women. In particular, women are more likely than men to blame themselves for their children’s problems and to see their identity threatened by their children’s illness [29]. As Simon noted, the differences do not just reflect differences in engagement with domestic responsibilities [71]. Even when men and women experience the same conflicts regarding work and family roles, these conflicts are interpreted differently and often to the detriment of women [71].

Our second objective was to examine how access to healthcare services affect caregivers of people with rare diseases. The most frequently cited barrier to accessing healthcare was the difficulty in obtaining a (correct) diagnosis, or the so-called diagnostic odyssey. As Nutt et al. have previously noted, delays in diagnosis and misdiagnosis are a major problem and can lead to many avoidable hospitalisations and inappropriate treatments and tests [58].

Lack of knowledge of medical staff was cited by caregivers as the most common reason for delayed diagnosis, failure of treatment or denial of social services. This led to conflicting information about the diagnosis, misunderstandings [78], or inadequate and missing information [26]. Caregivers reported how difficult it was to find healthcare providers who knew about the disease or had information about treatment. This finding is similar to that of Pelentsov et al. who found that the most frequently cited need of parents of people with rare diseases was the need for information [20]. The lack of information available to parents makes this situation difficult to deal with. Unsurprisingly, parents felt that more information and a better understanding of the disease and what to expect would help them cope with the challenges [17]. If they knew what community health services were available for their child, they could plan more confidently for the future [62]. Caregivers criticise that they refuse to seek help to overcome the limits of their knowledge [35]. The lack of knowledge and treatment options makes caregivers feel that they have to take whatever they can get, even if the treatment is not approved.

Caregivers reported limited collaboration and integration between healthcare providers, prompting them to take on the role of care coordinator to ensure that all healthcare providers have information and the newest results. They must advocate for the person they are caring for has access to much-needed services. As McMullan et al. found, caregivers often have unparalleled personal knowledge of how a rare disease affects the person, although they rarely receive enough practical or medical information to help them in their role [50]. This phenomenon often disrupts the relationship between caregivers and service providers, with caregivers taking on the role of “expert” [17]. The lack of involvement of many different service providers, but particularly the lack of involvement of healthcare providers, could be due to uncertainty about their knowledge of the rare diagnosis and therefor their suitability for treatment [44].

Problems with access to health care services also affect the financial resources of caregivers as they have to pay out of their own pocket. Although the government sometimes provides a small amount in the form of a caregiver allowance, this is not nearly enough to cover the costs of medical treatment and travelling [3]. Raising a disabled child comes with significant additional costs [50], and sometimes caregivers have to reduce their paid working hours or leave the workforce altogether [63]. Another obstacle to the utilisation of care services is the lack of insurance coverage in certain countries because insurance companies are not aware of the rare disease. A study by Gater et al. found that many drugs that are potentially effective for rare diseases are not covered by health insurance companies when used off-label in rare diseases patients [27].

We found that caregivers were able to overcome the barrier to accessing health care by interacting with other caregivers or participating in support groups, which were valuable sources of information.

Our third objective was to examine whether access to health care services affects the QOL of caregivers of people with rare diseases. Similar to our findings, Spencer-Tansley has found factors that affect the QOL, such as caregivers having to assume the role of care coordinator, social isolation, additional financial burden, and lower QOL due to time spent on care [72]. Challenges related to access to and coordination of services negatively impacted mental health. These included: trying to access health services or treatments, how care is coordinated, access to financial support, and access to other supports such as social care or respite care. Thus, we can assume that poor coordination of health services poses many emotional challenges. We found that challenges to accessing health services affect the psychological and social dimensions of QOL. Delays and difficulties in diagnosis and treatment and misdiagnosis were associated with anxiety, frustration, and stress, which affected the psychological dimension.

Even when the disease is diagnosed, finding a competent specialist can be a major problem. The psychological dimension of caregivers’ QOL is also affected, as caregivers face obstacles due to the rarity of the disease and are confronted with the lack of knowledge of healthcare providers. As von der Lippe et al. have found, the lack of knowledge about the rare disease can lead to delayed diagnosis, incorrect treatment or denial of services, all of which can have a negative impact on caregivers’ QOL [78]. Spencer-Tansley has also found that interactions with healthcare providers have a negative impact on mental health [72], and usually lead to stress, frustration and anxiety [17, 63]. Many caregivers report that physicians are confronted with the disease for the first time and have no treatment plan for the disease, that they have no information about possible support groups, and that caregivers usually have more information than providers. Because of this, caregivers feel more responsible and have emotional reactions such as loneliness and insecurity related to the social dimension of QOL. An additional stressor for caregivers is the lack of involvement of healthcare providers. This is also a reason why they feel abandoned and frustrated, which affects both the psychological and social dimensions of QOL. The difficulty of finding a provider may be complicated by the fact that rare diseases can affect multiple organ systems. The number of specialist clinics is limited and they are located in regional centres [4] and can therefore be far away, requiring caregivers to travel long distances. This can place an additional strain on caregivers’ financial resources. Previous studies of thyroid cancer survivors also suggest that financial hardship and negative financial events are associated with poorer QOL [55].

We found that caregivers who connected with other peers online had access to information and emotional support. This is consistent with previous research on breast cancer patients that both social support from other patients can improve QOL [48].

A recurring observation in all countries analysed in this review is the predominant involvement of mothers as primary caregivers. Regardless of cultural context, mothers were the main caregivers, indicating a universal caregiving role. This finding is consistent with the comprehensive cross-cultural study by Weisner and Gallimore, who analysed data from 186 societies worldwide and found that mothers, along with female adult relatives and female children, predominantly assumed the role of primary caregiver for infants and young children [80].

Studies from Germany [13], Spain [12], Australia [56], Canada [49], Brazil [31] and Italy reported lower levels of social support, which may mean that participants from these Western countries, known as more individualistic cultures, may prioritise personal autonomy over collective caregiving tasks or may simply not have the ability to rely on family due to their schedules. This observation is consistent with the findings of Humphrey and Bliuc, who found that while individualistic traits such as personal fulfilment and freedom of expression can enhance psychological well-being, other aspects of individualism such as limited social support, competitiveness and social comparison may contribute to a decline in social relationships and mental health in Western populations in recent decades [37]. This could also explain the reliance on formal care services instead of family support mentioned in the Australian (Mori et al., 2017) and Canadian [22] studies could indicate cultural norms or societal structures that favour formalised care services over informal support networks, or simply the availability of these services that have yet to be developed in other countries.

In contrast, Nigerian caregivers reported a medium to high levels of perceived social support, indicating a more supportive social environment for caregivers in this cultural context [1]. Cultural factors such as strong family ties, community support networks or cultural norms that emphasise collective responsibility for caregiving may contribute to the higher levels of social support observed among Nigerian caregivers. In Nigeria, as in many other African countries, social support from the extended family is taken so much for granted that it is commonly referred to as the “African extended family system” [23].

This review compiled detailed information on the impact of caring for people with rare diseases on caregivers’ QOL, with a particular focus on their experiences of accessing healthcare services for the people with rare diseases they care for. It is clear that, caregivers of people with rare diseases face many unique issues and should be better supported to alleviate their burden. This study shows that healthcare systems need new strategies, as the current healthcare systems often leave it up to caregivers to become rare disease experts and advocate for to access to treatment and help. This responsibility should not be left in the hands of caregivers but, needs to be addressed systematically. By highlighting the impact of barriers to accessing healthcare services, we wanted to encourage policy makers and care providers to develop new strategies to support caregivers and improve health outcomes.

Furthermore, the consistency of our scoping review with previous studies by Boettcher et al. and Pelentsov et al. [14, 62] emphasises the consistency of the challenges faced by caregivers in different contexts. While Pelentsov et al. focused primarily on the needs of parents of children with rare diseases, the parallels that emerge from their findings are strongly consistent with the challenges we identified in our study. This consistency highlights the universal nature of the challenges of caring for people with rare diseases and emphasises the need for comprehensive support systems tailored to the specific needs of carers around the world.

### Study limitations and strengths

Our study has some potential limitations. First, the search strategy was limited to English-language studies. Therefore, there may be other literature that is equally relevant to the area of QOL and access to health services but may have been overlooked. Although the inclusion of articles in other languages would likely increase the selection of relevant articles, the scientific world tends to publish as much as possible in a single (English) language. The selection of articles was primarily made by one reviewer, but in cases of doubt an independent review was conducted. Also, due to the search strategy, we may have excluded many rare diseases, so QOL and access to healthcare may not be well represented for all diseases. However, this limitation was addressed by a manual search of the reference lists of included studies, which allowed us to access many studies published under other search terms.

We found that in most of the studies we examined, the voices of female caregivers were present. Less is known about the experiences and challenges faced by male caregivers. The literature often emphasises the perspective of female caregivers, so we do not understand how male carers manage their role, cope with stress and interact with healthcare systems. We do not assume that caregivers are homogeneous. Therefore, caregiving and its relationship to gender and coping must be adequately assessed to provide an accurate description of the differences between men and women in relation to this phenomenon.

The included studies that used a qualitative design utilised purposive sampling, i.e., recruitment of participants focused on sources where caregivers of children with rare diseases were active, such as hospitals and rare disease support groups. This meant that participation was limited to those who had more connections to services and peer support. Therefore, it is possible that other perspectives and experiences of services were not well represented.

On the other hand, the study also has some important strengths. As far as we know, this is one of the few studies that examines access to health services among rare disease caregivers, focuses on caregivers’ quality of life, and examines how QOL is affected through the lens of barriers to accessing healthcare. This review also includes all known research in selected bibliographic databases. The strengths of this literature review lie in its methodological and systematic approach, that explores the experiences of caregivers of people living with rare diseases from both qualitative and quantitative perspectives. The review provides insights into the complexities of caring for people with rare diseases and highlight many areas for improvement in the future to enable better planning of health care and other services.

### Implications for practice

These findings may help to understand the problems associated with caring for people with rare diseases. This information could help service providers to better understand and help caregivers of people with rare diseases to appropriate support. It can help primary care physicians by providing information about the needs of caregivers, such as the need for continuity in dealing with diagnostic uncertainty and the provision of an empowering and collaborative approach.

The review emphasises the importance of prioritising carers of people living with rare diseases in Slovenia, as they face very different challenges to carers of older people, who already receive more attention [15, 60].

### Future research

Future research should focus on examining cultural or regional differences in the impact of rare diseases on caregiver QOL and access to healthcare. Further research could focus on examining the impact of rare diseases on caregiver’s QOL with treatable diseases comapred to those without treatment. It might also be interesting to examine at how the needs of caregivers change over time. Further research should also consider the use of longitudinal studies and larger samples to investigate the impact of care on QOL. Studies such as the one by Rotar-Pavlič et al. study on the experiences and feelings of informal caregivers of elderly in Slovenia and the challenges and difficulties they face in the society [60] would also be welcome.

## Conclusion

Caregivers’ QOL is impaired compared to parents with healthy children, parents of children with chronic diseases, and compared to normative values. Female caregivers appear to be more affected than male caregivers. We found that the physical, psychological, and social dimensions of caregivers’ QOL are most affected. Many factors seem to influence caregivers’ QOL. Caregivers of people with rare diseases have many common experiences in accessing healthcare. They struggle with lack of information, diagnosis, misdiagnosis, access to services, lack of engagement with healthcare providers and lack of treatment options. Barriers to accessing health care appear to affect caregivers’ QOL. Delays in diagnosis and lack of information can lead to increased anxiety and stress, which in turn can affect caregivers’ QOL, particularly the psychological and social dimensions. Peer support appears to be a great help in obtaining information about health services and provides emotional support. In summary, our findings reveal consistent patterns in the QOL of caregivers across different rare disease diagnoses and healthcare systems and highlight that caregivers of people with rare diseases face common challenges in different contexts.

### Electronic supplementary material

Below is the link to the electronic supplementary material.

Supplementary Material 1

## Data Availability

Not applicable.

## Abbreviations

QOL: Quality of life
