# Supplementary material for: Experiences of quality of life and access to health services among rare disease caregivers: a scoping review
Source: Orphanet J Rare Dis. 2024 Aug 31;19:319. doi: 10.1186/s13023-024-03327-2 (PMC11365242; doi:10.1186/s13023-024-03327-2)
Supplement: Supplementary file 1 — Supplementary Material 1 [file 13023_2024_3327_MOESM1_ESM.docx]

# **Additional files**

- 1. *Additional file* 1 – Details of selected articles found in the literature search on QOL of rare disease caregivers.

| **Author, year, country of origin** | **Study objective** | **Study design, study methods** | **Sample size, age and sex distribution** | **Type of rare disease** | **General themes** | **Study findings** |
| --- | --- | --- | --- | --- | --- | --- |
| Boettcher et al  2020  Germany  (19) | To examine the quality of life, mental health and associated protective factors of mothers and fathers caring for children with rare diseases requiring mechanical long-term ventilation. | Cross-sectional design;  The Ulm Quality of Life Inventory for Parents (ULQIE);  The Brief Symptom Inventory (BSI);  The Coping Health Inventory for Parents (CHIP);  The Oslo-Social Support Scale (OSSS-3);  Family Assessment Measure (FAM); | 110 parents including 72 mothers and 38 fathers; The age of the parents of the rare disease-afflicted children was significantly lower in mothers (M = 40.1, SD = 7.38) than in fathers (M = 43.1, SD = 7.38). | Severe disease classified as rare, currently requiring mechanical ventilation or potentially requiring mechanical ventilation | Quality of life in parents of children with rare diseases,  parental mental health,  parental overall use of coping mechanisms,  caregiver burden between mothers and fathers. | There are significant differences between mothers and fathers of children with rare diseases who require long-term mechanical ventilation in terms of their QoL and mental health. Mothers seem to be more negatively impacted by their child's disease. Caring for a child with a rare disease can be seen as a constant stressor in life. Psychosocial support services should therefore aim at strengthening the family situation, overcoming social isolation, strengthening intra-familial relationships and reinforcing coping strategies for the handling of the child's disease. |
| Berrocoso et al  2020  Spain  (20) | To characterize the sociodemographic and psychosocial profile of WHS caregivers and analyze how these variables impact their quality of life (QoL) and well-being. | Cross-sectional study;  WHOQOL-BREF  WHOQOL-SRPB;  Zarit Burden Interview – ZBI;  The Symptom Checklist-90-R - SCL-90-R;  Coping Strategies Inventory – CSI;  Social Network Questionnaire – SNQ. All questionnaires were spanish versions. | 22 caregivers – mean age 39.73;  19 female caregivers and 3 male caregivers | Wolf-Hirschhorn Syndrome | Sociodemographic and clinical profile of caregivers of children and young people with WHS in Spain;  Psychosocial and clinical characteristics of WHS caregivers | Caregivers of children with WHS received higher scores for anxiety about their loved one's future. Caregivers may have difficulty delegating care to other family members or support persons, which distances them from their social networks and adds to their emotional distress. Compared to a sample of the healthy population, caregivers show more symptoms of depression, somatization, obsessive-compulsive disorder, and hostility. WHS caregivers exhibited lower quality of life than caregivers of other rare, chronic diseases and normative reference populations. And higher or equal levels of symptoms than normative and primary caregiver populations. Caregiver burden has been shown to affect caregiver resources and outcomes. Considering that caregiver QoL was more favorable than the use of adaptive coping strategies and disengagement strategies, it showed a strong association with caregiver psychological distress. |
| Kim et al  2010  Korea  (21) | To investigate caregiver burden and health-related quality of life of mothers of children with mitochondrial disease, compared with those of mothers of children with intractable epilepsy. | Cross-sectional study;  The Zarit Burden Inventory;  Medical Outcomes Short Form 36;  Short version of the World Health Organization Quality of Life;  Beck Depression Inventory;  Beck Anxiety Inventory | 33 participants, all female, mean age 37.72 | Mitochondrial disease | Caregiver burden and health-related quality of life in mothers of children with mitochondrial disease in Korea. | Mothers of children with mitochondrial disease had significantly higher caregiver burden and poorer health-related quality of life, particularly in terms of role limitations, vitality, and mental health. They also had higher levels of depression and anxiety. Caregiver anxiety was the most important factor influencing overall caregiver burden. In mitochondrial diseases, the possibility of maternal inheritance and lack of information about the disease could increase anxiety and ultimately caregiver burden. Accurate information about rare diseases provided to caregivers could help reduce their anxiety. |
| Mori et al  2017  Australia  (22) | To examine primary caregivers’ wellbeing and family quality of life among families with a child living with the CDKL5 disorder, and determine the relationships with a range of factors from child’s characteristics through family circumstances to availability of public resources. | Cross-sectional study;  The Short Form 12 Health Survey Version 2;  The Beach Center Family Quality of Life Scale | 131 (87.9%) biological mothers, 16 (10.7%) biological fathers, one foster mother (0.7%) and one grandparent;  Mean age 38.2 | CDKL5 disorder | Primary caregiver wellbeing;  Family quality of life | Emotional well-being was significantly impaired in this group of caregivers and was particularly related to more severe child sleep problems and family financial difficulties. Family QoL was generally rated lowest among those who used respite care extensively, suggesting that these families may be more stressed by daily caregiving. |
| Antoniadi et al  2021  Ireland  (23) | To identify caregivers whose quality of life (QoL) may be impacted as a result of caring for a person with ALS. | Cohort study;  Edinburgh Cognitive and Behavioural ALS Screen (ECAS);  Beaumont Behavioural Inventory (BBI);  Hospital Anxiety and Depression Scale (HADS)  Zarit Burden Interview (ZBI);  McGill Quality of Life questionnaire (MQoL) | 90 patients and their caregiver  ALS patients had a mean age of 64.8 (The percentage of male patients was 59% (53M, 37F).  The majority of caregivers were female (63 female carers, 70%) and the average caregiver age was 55.5 years of age. | Amyotrophic Lateral Sclerosis | Identification of predictors of caregiver QoL;  Modelling caregiver QoL for clinical usage | Existential quality of life and caregiver burden, as well as patient depression and pre-symptom employment, were the characteristics that had the greatest impact on predicting caregiver quality of life. The study found a relationship between caregiver QoL and feelings of burden and depression. |
| Qi et al  2021  China  (24) | To describe the health care service utilization through participants’ perspective, estimated the cost of illness, health-related quality of life of patients with GD, and the quality of life of their caregivers. | Cohort retrospective study;  An online retrospective survey:  SF-36  Zarit Burden Inventory;  Social Support Rating Scale;  Pittsburgh Sleep Quality Index | 40 patients and 49 caregivers | Gaucher disease | Health service utilization;  Cost of illness;  Social support and health-related quality of life of patients and their caregivers | Patients with GD in China often have the frustrating experience of a high misdiagnosis rate, long delayed diagnosis, significant cost, and deteriorated health-related quality of life. For patients with GD, a rapid, accurate diagnostic tool for earlier and timely definitive diagnosis of GD is urgently needed. Measures to improve the availability and affordability of orphan drugs need to be developed holistically in China. Raising awareness of early signs/symptoms or less severe manifestations is critical to achieve earlier diagnosis and timely treatment of rare diseases, reduce burden on caregivers, and provide much needed social support. |
| Wu et al  2020  Australia  (25) | To quantify the parental health spillover effects across four groups of paediatric rare genetic conditions. | Cross-sectional study;  Short-Form Health survey (SF-12 v2);  Child Health Utility 9D survey (CHU9D) | 207 parents;  88,1% female  Mean age: 38,4 | Genetic kidney disease  Mitochondrial disease  Epileptic encephalopathy  Brain malformation | Parental health spillover effect | There is a significant health spillover effect associated with rare genetic diseases. Having a child with a rare genetic disorder has been associated with a reduction in parental HRQoL. There is a positive association between the QoL of parents and the QoL of their children. |
| Xu et al  2021  China  (26) | To assess HRQoL and related factors among family caregivers of patients with Gaucher disease in China. | Cross-sectional study;  The 36‐Item Short‐Form Health Survey Questionnaire (SF‐36);  Zung's Self‐Rating Anxiety Scale(SAS):  Zung's Self‐Rating Depression Scale (SDS);  Multidimensional Scale of Perceived Social Support (MSPSS);  Herth Hope Index (HHI) | 49 participants  36 female, 13 male  Mean age: 44,04 | Gaucher disease | HRQoL of family caregivers for Gaucher disease patients;  Factors associated with HRQoL of family caregivers | Family caregivers of patients with GD in China report low HRQoL in all eight SF -36 domains. Gender, education, daily caregiving time, anxiety, and perceived illness severity are significant predictors of physical component scores. Significant predictors of mental component scores are help from others with caregiving, anxiety, perceived severity of illness, and type of health insurance. |
| Shapiro et al  2019  USA  (27) | To provide clinical advisors with the necessary information to assist them in with their deliberations and recommendations to better understand families' experiences of caring for patients with Sanfilippo B.  To measure clinical perceptions of caregiver burden, understand clinicians' perspectives on the extent of caregiver stress and burden, and identify priorities for caregiver treatment and support. | Mixed methods design;  Sequential exploratory design;  Telephone interview;  Online survey; | 26 parents caring for children with Sanfilippo B;  5 global clinical experts in Sanfilippo B;  46 Sanfilippo clinicians and patient advocates | Sanfilippo B syndrome | The impact of caregiver burden on the QoL of Sanfilippo B families; Mitigating disease burden | To alleviate caregiver burden, a wide range of Sanfilippo B-specific interventions and support services should be provided that not only address the patient's behaviors and symptoms, but also address the changing needs of family caregivers as the disease progresses and support the mental and physical health needs of family caregivers. Carer burden is dynamic and is influenced by the patient's age and stage of illness. As a result, caregiver burden remains high throughout the patient's life, with caregivers experiencing a disease-specific and predominantly negative impact on QoL. |
| Rodríguez Bermejo et al  2021  Spain  (28) | To evaluate the costs of informal care for children with neuromuscular disease and evaluate how physical and psychological health is associated with socio-demographic variables. | Cross sectional study;  The questionnaire of economic costs;  The PHQ-15;  The Zarit Overload Scale;  The Satisfaction with Life Scale (SWLS);  CarerQol;  The Barthel Index | 110 participants;  19 male (17.2%),  91 female (82.8%);  Mean age: 46,04 | Neuromuscular Disease | Social and Health Costs; Physical and Mental Costs;  Percentage of Family Income; Cost of Informal Care per Person with NMD;  Work Status | Caregivers of children with NMD face a number of high costs related to their own physical and psychological health. Children with severe support needs, as well as their caregivers carers, had greater needs. |
| Landfeldt et al  2016  Sweden  (29) | To estimate the caregiver burden associated with Duchenne muscular dystrophy. | Cross-sectional study;  EuroQol EQ-5D-3L (EQ-5D);  Visual Analogue Scale (VAS);  SF-12 Health Survey (SF-12); | 770 participants;  609 (79%) female;  Mean age: 44 | Duchenne muscular dystrophy | Prevalence of anxiety and depression;  Caregiver health-related quality of life;  Subjective caregiver burden; | Caring for a person with DMD can be associated with a substantial burden and markedly impaired HRQoL. Caregivers indicated being moderately or extremely anxious or depressed. Mental health summary score from the SF-12 was significantly lower than general population reference data. Caregivers to patients in fair/poor compared to excellent health had a sixfold risk increase of anxiety and depression. Employment outside of the home is a predictor of stress in caregivers |
| McMillan et al  2021  Canada  (30) | To characterize the burden of Spinal Muscular Atrophy (SMA) in Canada as reported by patients and caregivers, including disease and treatment impacts, indirect costs, and caregiver burden. | Cross-sectional study;  EQ-5D-5L;  Caregiver Strain Index (CSI);  Visual analogue scale (VAS). | 965 patients; 387 female (40.2%) and 576 male (59.8%), mean age: 13.70;  952 caregivers  551 female (57.6%), 406 male (42.4%), mean age: 35.25 | Spinal Muscular Atrophy (SMA) | Healthcare resource use; Quality of life; Caregiver-related burden; Caregiver strain | SMA affects the quality of life of individuals diagnosed with the disease and their caregivers, as measured by the EQ -5D-5L. Other impacts include reduced attendance at work or school, increased need for support at work or school, significant SMA -related consumption of health care resources and travel to appointments, and costs associated with housing adaptations and assistive devices. Unpaid caregivers of individuals with SMA report significant caregiving-related burden, as demonstrated by the number of caregivers reporting changes in personal plans, disturbed sleep, and adjustments to work. Other aspects of caregiver health that caregivers reported being affected by caring for a person with SMA included receiving respite care for exhaustion and undergoing physiotherapy for an injury related to lifting/transferring the patient. |
| Kanters et al  2013  Netherlands  (31) |  | Cross-sectional study;  The CarerQol; CarerQol-VAS; Self-rated burden scale; EuroQol-5D | 120 patients, 88 (73%) were recipients of informal care;  67 informal caregivers | Pompe disease | Burden of caregiving;  Well being of caregiver; Happines derivedfrom providing care | Informal caregiving of patients with Pompe's disease causes burden to the informal caregiver. Problems in daily activities due to informal caregiving are present in 50% of informal caregivers and 40% of caregivers have psychological and/or mental problems. Despite the negative aspects of informal caregiving, it is important to emphasize that the majority of caregivers derive fulfillment from caregiving and that, on average, caregivers derive utility from providing informal caregiving of patients with Pompe disease. |
| Mengel et al  2021  Germany  (32) | To explore the impact of NPC on patients’ and caregivers’ daily lives to understand the burden of disease. | Mixed methods study;  Sequential explanatory design;  Online survey;  Telephone interview | 49 patients completed online survey; 28 patients completed telephone interview; 43 caregivers completed online survey; 23 caregivers completed telephone interview | Niemann-Pick disease type C (NPC) | Importance of symptoms; Impact on patients and caregivers; Impact of NPC by degree of disability; Impact of NPC by age; Impact of NPC by age of symptom onset | Activities of daily living and HRQOL were impaired in the majority of patients and their caregivers, regardless of current age or age of onset of symptoms. The impact of NPC on patients and caregivers depended on the level of disability, with greater impact at higher levels of disability. |
| Guarany et al  2015  Brasil  (33) | To evaluate the  QoL of caregivers of patients with Mucopolysaccharidosis (MPS). | Cross-sectional study;  WHOQOL-BREF questionnaire | 11 mothers;  Median age: 35 years | Mucopolysaccharidosis | Quality of life and different types of MPS. | Caregivers of patients with  MPS experience low QoL, with caregivers of patients with  MPS II experiencing the poorest quality. |
| Witt et al  2019  Germany  (34) | To investigate the quality of life of children with achondroplasia from child- and parent perspective as well as the parental quality of life. | Cross-sectional study;  PedsQL 4.0™ questionnaire;  Short-Form-8 questionnaire (SF-8) | 73 children, 37 male, 36 female, 73 parents; 17 male, 56 female | Achondroplasia | Children’s quality of life; Parental quality of life; Correlations between children’s quality of life and parental quality of life | The decreased QoL of children with achondroplasia can be explained by the physical limitations and impairments as well as the various challenges in daily life reported by the children. Correlations between children's parent-reported quality of life and parental QoL showed significant results for both parental physical QoL and parental mental QoL. The higher the parental QoL, the higher the parent-reported QoL of the children. This is also shown by the correlation between parental physical QoL. The reduced parental mental QoL refers to the burden on the parents that can result from the child's chronic health condition. |
| Ahanotu, Ibikunle and Hammed  2018  Nigeria  (35) | To investigate the burden of caregiving, social support and quality of life of informal caregivers of patients with  Cerebral palsy. | Cross-sectional study;  World Health  Organization Quality of Life Bref (WHOQOL-Bref); Bref (WHOQOL-Bref). Also, social support was assessed using Multidimensional Scale of Perceived Social Support (MSPSS); Caregivers Strain Index (CSI) | 78 informal caregivers; 22 males and 56 females; Mean  age of 36.54 | Cerebral palsy | Burden of caregiving; Social support; Quality of life o | Caring for a child with CP had significant impact on the level of burden, social support and quality of life of informal caregivers. The informal caregiver had a significant level of burden, a moderate of social support mainly from family and significant others and a high level of quality of life. |
| Feeley et al  2014  USA  (36) | To explore if sleep quality, stress, and caregiver burden influence overall QOL in maternal caregivers. | Cross-sectional study;  The Pittsburgh Sleep Quality Index (PSQI);  The perceived stress scale (PSS); Montgomery-Borgatta Caregiver Burden Scale-Revised (MBCBS-R); WHO QOL-BREF; The Center for Epidemiological Studies Depression Scale (CES-D) | 61 maternal caregivers; Mean age 29.59 years | Bronchopulmonary Dysplasia | Sleep quality; Caregiver burden; Stress; | Sleep quality and depressive symptoms were significant predictors of QOL. Sleep quality was found to be the most significant predictor of quality of life in maternal caregivers. |
| Galvin et al  2020  Ireland  (37) | To explore individual quality of life of people with Amyotrophic Lateral Sclerosis (ALS) and their informal caregivers over time. | Mixed methods study;  Concurrent triangulation design;  Semi-structured interviews; The Hospital Anxiety and Depression Scale (HADS); Schedule for the Evaluation of Individual Quality of Life (SEIQoL-DW); The Zarit Burden Interview | 28 patients, 19 male, 9 female, mean age 61, 8 years; 28 caregivers, 9 male, 19 female, mean age 60,06 years | Amyotrophic Lateral Sclerosis (ALS) | Dyad characteristics;  Individual quality of life; Life areas contributing to individual quality of life; | Family, hobbies, and social activities were the most important self-defined factors contributing to quality of life. The importance of health decreased over time compared to other areas. Friends and finances became less important to patients, but were rated as more important by caregivers as the disease progressed. Psychological distress was higher among caregivers. Caregiver burden increased steadily. |
| Williams et al  2009  USA  (38) | To determine the needs of ALS family caregivers. | Mixed methods study;  Sequential transformative design;  Interviews;  SF-8  Concept Mapping | 19 caregivers | Amyotrophic Lateral Sclerosis (ALS) | Caregiver needs;  Quality of life | The SF-8 results indicated that caregivers who resided with their ALS family member have poorer mental and physical health than family caregivers who did not have primary residence with the ALS patient. |
| Roach et al  2009  USA  (39) | To examine QOL in ALS patients and their caregivers over the course of the illness. | Cohort study;  McGill Quality of Life Questionnaire; ALS Functional Rating Scale | 55 patients, 64% male and 36% female, mean age 58.4 years, 53 caregivers, 33% male and 67% female, mean age 56,02 | Amyotrophic Lateral Sclerosis (ALS) | Quality of life in patients and caregivers | Low QoL among ALS patients is likely due to preexisting individual differences, while both individual differences such as demographic characteristics (e.g., age) and disease progression are likely to influence caregiver QoL. The passage of time did not affect patients' QoL, but overall QoL, and especially QoL related to physical symptoms, decreased over time among caregivers. Patient and carer gender was largely unrelated to QoL, but younger caregivers had lower QoL in a number of domains. |
| Chio et al  2010  Italy  (40) | To evaluate the frequency of neurobehavioral symptoms related to FTLD in a consecutive series of amyotrophic lateral sclerosis (ALS) patients and to assess their influence on patients’ and caregivers’ mood, burden, and quality of life. | Cross-sectional study;  Frontal Systems Behavior Scale (FrSBe)  Mini Mental State Examination (MMSE);  Zung Depression Scale (ZDS); McGill Quality of Life Questionnaire (MQoL); Caregiver Burden Inventory (CBI); ALS-Functional Rating Scale (ALS-FRS) | 70 patients included 37 men and 33 women, mean age 61.9 years; 70 caregivers included 23 men and 47 women, mean age 54.7 years. | Amyotrophic Lateral Sclerosis (ALS) | Neurobehavioral symptoms, Caregivers’ strain and quality of life; | Neurobehavioral symptoms have a profound negative impact on caregivers’ psychological status and were highly related with caregivers’ burden. Neurobehavioral symptoms were related to the presence of bulbar symptoms at the time of the interview, but not to patients’ age, gender, or physical status (ALS-FRS score). Patients’ neurobehavioral symptoms were significantly related to lower caregivers’ quality of life, highest depression. |
| Alshubaili et al  2008  Kuwait  (41) | To compare the subjective QOL of family caregivers of persons with relapsing remitting and progressive MS, with those of a matched general population sample and caregivers of diabetes and psychiatric patients. To assess the relationship of QOL with caregiver attitudes to MS and patient's variables. | Cross-sectional study;  WHO QOL Instrument (WHOQOL-Bref);  Beck's Depression Inventory | 170 caregivers, 60 men and 86 women mean age 35.7 | Multiple sclerosis | Quality of life | Caregivers need specific attention if they are less educated, unemployed, afraid of having MS and caring for patients with longer duration of illness and less education. In particular, attention to patients' depression and disability could improve caregivers' QOL. Caregivers need specific programs to address fear of having MS, negative attitudes to illness and their unmet needs. |
| Minaya et al  2014  France  (42) | To analyze the impact of gliomas in caregivers' quality of life (QoL) and to compare this specific population to other oncology caregivers and the normative population in order to find differences and understand which aspects of QoL are more impacted. | Cross-sectional study;  CareGiver Oncology Quality of Life questionnaire (CarGOQoL) | 50 caregivers, 28% male, age 30–77 years. | Gliomas | Quality of life | Caregivers of patients with gliomas showed increased burden scores and lower scores for the leisure time dimension. This could be explained by their unique care situation, in which patients become more limited physically and cognitively. |
| Khan, Pallant and Brand  2007  Austrailia  (43) | To describe the level of caregiver strain and factors associated with caregiver self-efficacy and quality of life (QoL) in a community cohort with multiple sclerosis (MS). | Cohort study;  Caregiver Strain Index (CSI); Caregiver Strain Index (CSI); caregiver self-reported burden (SRB); Assessment of Quality of Life (AQoL); General Self Efficacy Scale questionnaire (GSES) | 62 informal caregivers, male 25, 37 mean age 54,2 years  62 patients, 40 female, 22 male, mean age 51,7 | Multiple sclerosis | Caregiver strain; Self-efficacy, Quality of life, Self-rated burden | Caregivers of persons with MS reporting high levels of caregiver strain experienced a lower QoL and were caring for persons with MS with a lower QoL and higher levels of depression and anxiety. |
| Szczepaniak-Kubat et al  2012  Poland  (44) | Objective assessment of the quality of life of parents of children with osteogenesis imperfecta (OI) and of its determinant factors. | Cross-sectional study;  WHOQOL-BREF; | 25 mothers, mean age 34.3 years; 24 fathers, mean age 38.1 years | Osteogenesis Imperfecta (OI) | Quality of life | The child's illness did not affect the respondents' globala ssessment of quality of life, their health, or their quality of life in terms of physical and mental status and social relationships. The parents of children with severe OI rated the area of life related to the environment in which they live worse than the parents of children with mild OI. Respondents' assessment of global quality of life did not depend on the socioeconomic status of the family and the help they received in caring for the child. |
| Gauthier et al  2007  Italy  (45) | To evaluate the modification of quality of life (QoL) and depression in a series of amyotrophic lateral sclerosis (ALS) patient-caregiver couples during a period of 9 months and compare them to patients' ALS Functional Rating Scale (ALS-FRS). | Cohort study;  Zung Depression Scale (ZDS); McGill Quality of Life Questionnaire (MQoL); Caregiver Burden Inventory (CBI); Self-Perceived Burden Scale (SPBS). | 31 patients, 21 male and 9 women, mean age 58.8 years, 31 caregivers, 9 male, 21 female, mean age 54.5 years | Amyotrophic Lateral Sclerosis (ALS) | Quality of life; Depression | Patients with amyotrophic lateral sclerosis experienced significant stabilisation of quality of life and depression over a 9-month period, whereas the burden and depression of their caregivers increased markedly. |

- 1. Additional file 2: Details of selected articles on access to health services of rare disease caregivers.

| **Author, year, country of origin** | **Study objective** | **Study design, study methods,** | **Sample size, age and sex distribution** | **Sample type** | **Type of rare disease** | **General themes** | **Study outcomes** |
| --- | --- | --- | --- | --- | --- | --- | --- |
| Currie and Szabo  2019  Canada  (46) | To explore perceptions and experiences of the medical and social supports  that exist for their families within their local communities. | Qualitative phenomenological study  An interpretive phenomenological approach  Semi-structured interview either face-to-face or telephone interview | 15 parents of children with rare disease | Purposive sampling | Rare  neurodevelopmental diseases | Lack of knowledge among health professionals;  Insufficient collaboration between different clinics and specialists treating a child with a rare disease;  Barriers to accessing health services;  Parents have to take different roles to access services. | The rarity of the disease is the cause of the complexity in obtaining care. There are significant gaps in health care management and support networks for families living with a chronic rare disease. |
| Hiremath, G. et al  2018  USA  (47) | Evaluate the usefulness of online platforms for accessing a dispersed EGID community through an online survey; to identify the EGID community's perceived unmet needs and barriers to health care in domains that are relevant to them; to explore whether perceived unmet needs and barriers differ between adult EGID patients and adult caregivers of children with EGID. | Delphi study  Sequential exploratory model  Focus groups, online survey | Focus group participants were: 6 adult EGID patients and 16 adult caregivers. The online survey participants were:  90 EGID patients and 271 adult caregivers. | Purposive sampling | EGID - eosinophilic gastrointestinal disorders | Barriers to health care;  Social aspects;  Emotional impact; | EGIDs are receiving more attention because they are better recognized in the health care system, the pathophysiology is better understood, there are advances in therapies on the market, and the public is better informed. However, family caregivers and patients continue to face a variety of challenges, including a lack of specialized care, delays in diagnosis, financial burdens, negative social consequences, psychosocial consequences, and emotional distress. |
| Kesselheim  2014  USA  (48) | To explore rare disease patients’, caregivers’, and advocates’ experiences with their conditions and the health care system and their perspectives on drug development. | Qualitative grounded theory study; Focus groups;  Grounded theory | 23 individuals (5 men and 18 women; 8 of them were caregivers; | Purposive sampling | Various rare diseases such as: tuberous sclerosis, phelan–McDermid syndrome, hemophilia, pulmonary artery hypertension... | Seeking Care;  Financial concerns and insurance coverage;  Psychosocial concerns; Being an advocate; Views on drug development and testing; | The lack of knowledge and treatment has serious implications for the health care, social lives, and emotional well-being of patients and their caregivers. The difficulty of finding knowledgeable medical care, the sense of isolation and lack of support, and the financial and social burdens they faced, combined with the limited availability of treatments, meant that they were willing to take risks with their treatment in the hope of some benefit. |
| Baumbusch, Mayer, Sloan-Yip  2018  Canada  (49) | To explore parents’ experiences of navigating the healthcare system for their child with a rare disease. | Qualitative interpretive phenomenological study; Interpretive description  Semi-structured interviews | 15 mothers, 1 father;  Mean age: 40 years | Snowball sampling | Included diseases were defined as rare by lifetime prevalence of 1 in 2000. | The diagnostic journey; Seeking and accessing services; Peer support | Parents found it difficult to find and obtain a diagnosis for their child and subsequently access services. Their information needs were not adequately addressed by GPs or specialists. Parents' experiences were particularly related to the provision of information at the time of diagnosis, barriers to accessing certain services and poor coordination of care. Issues around deficits in access and support from services led to increased advocacy from carers. Online contact with peers provided access to information and emotional support. |
| Cardinali, Migliorini and Rania  2019  Italy  (50) | To increase knowledge on the impact that rare disease could have on caregiver’s perception of daily life, feelings, behaviors and social support. | Qualitative grounded theory study; A semi-structured interview, | 15 parents, 7 fathers and 8 mother, mean age 52.12 | Convenience sampling | Aicardi syndrome , Angelman syndrome, Arginine succinic aciduria, Chromosome 22 Ring, Fryns syndrome, Goldenhar syndrome, Klinefelter syndrome, 49 XXXXY, Lesch-Nyhan syndrome, Mucolipidosis, type III, Prader-Willi syndrome, Rett syndrome | Common challanges, social support, | A common area of crisis that characterizes mothers and fathers concerns diagnostic research and difficulties in communicating with medical professionals, such as diagnostic delays, missing diagnoses, and misdiagnosis. Uncertainty about the future ​is compounded by the lack of information about the disease and its treatment. The multiplicity of professionals and services involved presents a fragmented framework that does not favor the necessary coordination between interventions, made more difficult by the unusual nature of the symptoms and the ambiguity of the diagnosis that often characterize rare diseases. This context reinforces the impression of a lack of information and coordination in the health system. The parents of a child with a rare disease must coordinate a series of basic activities for the management of the disease and work to obtain the correct diagnosis and the associated rights needed to access the various social support services and overcome bureaucratic difficulties. |
| Grut  2012  Norway  (51) | To contribute to the understanding of how people that live with a rare disorder experience their contact with service-providers when they access public health and welfare services, and discusses whether they have certain common experiences in thisregard that cut across the various disorders. | Qualitative interpretive phenomenological study;  Semi structured interviews; | 94 participants; 51 adults between 20 and 70 years of agewith a diagnosis; 33 parents of a child between the ages of 3 and 16 years with a diagnosis or of an adult with a diagnosis and reduced ability to give informed consent. | Purposive sampling | Congenital medical conditions | Experiences with healthcare providers | Service providers across a range of sectors and services seemed reluctant to become involved in situations that implied diagnoses they were unfamiliar with. Moreover, they seemed unwilling to accept information offered to them by users and reluctant to seek information themselves. When professionals intervened, they tended to basetheir judgments on their personal assumptions, resulting in incorrect responses. The negative responses of service providers represent a significant barrier to accessing appropriate services and support for people with rare diseases. |
| Čagalj, Buljevac and Leutar  2018  Croatia  (52) | To obtain insight into the experiences of mothers of children with PWS with the formal support system in Croatia. | Qualitative interpretive phenomenological study;  Semi strucutred interviews;  Thematic analysis | 5 mothers, mean age 44 years | Purposive sampling | Prader-Willi syndrome | Social services; Health care system; Living with rare disease | Formal support for mothers of children with PWS is characterised by a lack of knowledge and awareness about PWS, leading to a lack of empathy on the part of professionals and to their viewing children with PWS as those who cannot gain certain social rights. The findings indicate that mothers had to use their own strategies and skills to gain support. Although some parts of the findings suggest that support was better and more appropriate when PWS was finally diagnosed, it should be noted that mothers can only receive professional support if they travel to the capital. |

## *Addition file 3 – Search strategies:*

|  | PubMed Central | PubMed | Ovid Medline | Ebsco Cinahl |
| --- | --- | --- | --- | --- |
| Quality of life | ((((((rare diseases[MeSH Major Topic]) OR rare condition[Title]) OR rare disorder) OR orphan disease)) AND ((((caregiver[MeSH Major Topic]) OR carer[Title]) OR family caregiver[Title]) OR care giver[Title])) AND (((quality of life[MeSH Major Topic]) OR health related quality of life[Title]) OR HRQOL[Title]) | ((((((((((rare diseases[MeSH Major Topic]) OR (rare condition[Title])) OR (rare disorder[Title])) OR (orphan disease[Title])) AND (caregiver[MeSH Major Topic])) OR (carer[Title])) OR (family caregiver[Title])) OR (care giver[Title])) AND (quality of life[MeSH Major Topic])) OR (health related quality of life[Title])) OR (HRQOL[Title]) | ("rare diseases*" OR "rare condition*" OR "orphan disease*" OR "rare disorder*") AND (caregivers* OR carer* OR "family caregivers*" OR "care givers*") AND ("quality of life*" OR "health related quality of life*" OR HRQOL*)” | Rare diseases OR rare condtiion OR rare disorder OR oprhan disease AND caregiver OR carer OR family caregivers OR care giver AND qualiy of life OR health related quality of life OR hrqol |
| Access to health services | ((((((rare diseases[MeSH Major Topic]) OR rare condition[Title]) OR rare disorder) OR orphan disease)) AND ((((caregiver[MeSH Major Topic]) OR carer[Title]) OR family caregiver[Title]) OR care giver[Title])) AND (((access to healthcare[MeSH Major Topic]) OR availability of health services[Title]) OR access to health service[Title]) | ((((((((((rare diseases[MeSH Major Topic]) OR (rare condition[Title])) OR (rare disorder[Title])) OR (orphan disease[Title])) AND (caregiver[MeSH Major Topic])) OR (carer[Title])) OR (family caregiver[Title])) OR (care giver[Title])) AND (access to healthcare[MeSH Major Topic])) OR (availability of health services[Title])) OR (access to health services[Title]) | ("rare diseases*" OR "rare condition*" OR "orphan disease*" OR "rare disorder*") AND ("caregivers*" OR "carer*" OR "family caregivers*" OR "care givers*") AND ("access to healthcare*" OR "availability of health services*" OR "access to health services*") | Rare diseases OR rare condtiion OR rare disorder OR oprhan disease AND caregiver OR carer OR family caregivers OR care giver AND access to healthcare OR availability of health services OR access to health services |

## *Additional file 4– Inclusion and exlusion criteria*

Inclusion criteria:

- Articles in written in English.
- Articles addressing the quality of life of informal caregivers caring for a person with a rare disease (as defined by the European Commission (< 5:10 000)).
- articles published between 2005 and 2021.
- Studies that address quality of life as an outcome of care for people with rare diseases and use standardized, validated questionnaires.
- Articles on informal caregivers of people with rare diseases and on parents, spouses, or siblings of people with rare diseases who are their primary caregivers.
- Articles in which the characteristic comparison condition was defined as parents of healthy children, general population norms, or parents of children with chronic diseases.
- Mixed methods or qualitative data on caregiver quality of life to gain greater insight into the impact on quality of life.
- Studies that examine access to health services using standardized, validated. questionnaires, as well as qualitative studies that describe access to health services.

Exclusion criteria:

- Exclusion of clinical trials of medications, surgical interventions, and psychosocial and health measures that affect quality of life.
- Validation studies of instruments measuring quality of life.
- Articles not written in English.
- Articles about caregivers of people with rare diseases and other family members who do not directly care for people with rare diseases, as well as formal caregivers of people with rare diseases.
- Articles when the disease is not considered rare.
- Articles that are considered too obscure: studies whose study population is unclear; studies with unclear or inadequate descriptions of their research methods; studies that don't clearly present the results or if the data are difficult to access or interpret; studies that are not directly relevant to the research question or objectives of the scoping review.
- Articles that are not original research.

## *Additional file 5 – A list of the included studies and the countries of origin:*

| **Countries** | **Number of studies:** | **Reference** |
| --- | --- | --- |
| Norway | 1 | Grut L, Kvam MH. Facing ignorance: people with rare disorders and their experiences with public health and welfare services. Scandinavian Journal of Disability Research 2013; 15:20-32. |
| Italy | 3 | Cardinali P, Migliorini L, Rania N. The Caregiving Experiences of Fathers and Mothers of Children With Rare Diseases in Italy: Challenges and Social Support Perceptions. Frontiers in psychology 2019; 10:1780-. |
|  |  | Chio A, Vignola A, Mastro E, Giudici AD, Iazzolino B, Calvo A, Moglia C, Montuschi A. Neurobehavioral symptoms in ALS are negatively related to caregivers' burden and quality of life. Eur J Neurol 2010; 17:1298-303. |
|  |  | Gauthier A, Vignola A, Calvo A, Cavallo E, Moglia C, Sellitti L, Mutani R, Chió A. A Longitudinal Study on Quality of Life and Depression in ALS Patient–Caregiver Couples. Neurology 2007; 68:923-6. |
| USA | 6 | Shapiro E, Lourenco C, Mungan N, Muschol N, O’Neill C, Vijayaraghavan S. Analysis of the caregiver burden associated with Sanfilippo syndrome type B: panel recommendations based on qualitative and quantitative data. Orphanet Journal of Rare Diseases 2019; 14. |
|  |  | Feeley C, Turner-Henson A, Christian B, Avis K, Heaton K, Lozano D, Su X. Sleep Quality, Stress, Caregiver Burden, and Quality Of Life in Maternal Caregivers of Young Children With Bronchopulmonary Dysplasia. Journal of pediatric nursing 2013; 29. |
|  |  | Williams M, Donnelly J, Holmlund T, Battaglia M. ALS: Family caregiver needs and quality of life. Amyotrophic lateral sclerosis : official publication of the World Federation of Neurology Research Group on Motor Neuron Diseases 2008; 9:279-86. |
|  |  | Roach AR, Averill AJ, Segerstrom SC, Kasarskis EJ. The Dynamics of Quality of Life in ALS Patients and Caregivers. Annals of Behavioral Medicine 2009; 37:197-206. |
|  |  | Hiremath G, Kodroff E, Strobel M, Scott M, Book W, Reidy C, Kyle S, Mack D, Sable K, Abonia P, Spergel J, Gupta S, Furuta T, Rothenberg M, Dellon E. Individuals affected by eosinophilic gastrointestinal disorders have complex unmet needs and frequently experience unique barriers to care. Clinics and Research in Hepatology and Gastroenterology 2018; 42. |
|  |  | Kesselheim A, McGraw S, Thompson L, O'Keefe K, Gagne J. Development and Use of New Therapeutics for Rare Diseases: Views from Patients, Caregivers, and Advocates. The patient 2014; 8. |
| Poland | 1 | Szczepaniak-Kubat A, Kurnatowska O, Jakubowska-Pietkiewicz E, Chlebna-Sokół D. Assessment of Quality of Life of Parents of Children with Osteogenesis Imperfecta. Advances in clinical and experimental medicine : official organ Wroclaw Medical University 2012; 21:99-104. |
| Australia | 3 | Mori Y, Downs J, Wong K, Anderson B, Epstein A, Leonard H. Impacts of caring for a child with the CDKL5 disorder on parental wellbeing and family quality of life. Orphanet Journal of Rare Diseases 2017; 12. |
|  |  | Wu Y, Al-Janabi H, Mallett A, Quinlan C, Scheffer I, Howell K, Christodoulou J, Leventer R, Lockhart P, Stark Z, Boughtwood T, Goranitis I. Parental health spillover effects of paediatric rare genetic conditions. Quality of Life Research 2020; 29. |
|  |  | Khan F, Pallant J, Brand C. Caregiver strain and factors associated with caregiver self-efficacy and quality of life in a community cohort with multiple sclerosis. Disability and rehabilitation 2007; 29:1241-50. |
| France | 1 | Minaya P, Berbis J, Chinot O, Auquier P. Assessing the quality of life among caregivers of patients with gliomas. Neuro-oncology practice 2014; 1:191-7. |
| Kuwait | 1 | Alshubaili A, Ohaeri JU, Awadalla A, Mabrouk A. Family caregiver quality of life in multiple sclerosis among Kuwaitis: A controlled study. BMC Health Services Research 2008; 8. |
| Ireland | 2 | Antoniadi A, Galvin M, Heverin M, Hardiman O, Mooney C. Prediction of caregiver quality of life in amyotrophic lateral sclerosis using explainable machine learning. Scientific Reports 2021; 11:12237. |
|  |  | Galvin M, Gavin T, Mays I, Heverin M, Hardiman O. Individual quality of life in spousal ALS patient-caregiver dyads. Health and Quality of Life Outcomes 2020; 18. |
| Nigeria | 1 | Ahanotu C, Ibikunle P, Adebisi H. Burden of caregiving, social support and quality of life of informal caregivers of patients with cerebral palsy. Turkish Journal of Kinesiology 2018; 4:58-64. |
| Germany | 3 | Boettcher J, Denecke J, Barkmann C, Wiegand-Grefe S. Quality of Life and Mental Health in Mothers and Fathers Caring for Children and Adolescents with Rare Diseases Requiring Long-Term Mechanical Ventilation. International Journal of Environmental Research and Public Health 2020; 17:8975. |
|  |  | Mengel E, Patterson M, Chladek M, Guldberg C, i Dali C, Symonds T, Lloyd-Price L, Mathieson T, Crowe J, Burbridge C. Impacts and Burden of Niemann pick Type-C: a patient and caregiver perspective. Orphanet Journal of Rare Diseases 2021; 16. |
|  |  | Witt S, Kolb B, Bloemeke J, Mohnike K, Bullinger M, Quitmann J. Quality of life of children with achondroplasia and their parents - a German cross-sectional study. Orphanet Journal of Rare Diseases 2019; 14. |
| Brasil | 1 | Guarany N, Vanz A, Wilke M, Bender D, Borges M, Giugliani R, Schwartz I. Mucopolysaccharidosis: Caregiver Quality of Life. Journal of Inborn Errors of Metabolism & Screening 2015; 3. |
| Netherlands | 1 | Kanters T, Ans T, Brouwer W, Hakkaart- van Roijen L. The impact of informal care for patients with Pompe disease: An application of the CarerQol instrument. Molecular genetics and metabolism 2013. |
| Sweden | 1 | Landfeldt E, Lindgren P, Bell C, Guglieri M, Straub V, Lochmuller H, Bushby K. Quantifying the burden of caregiving in Duchenne muscular dystrophy. Journal of Neurology 2016; 263. |
| Spain | 2 | Berrocoso S, Amayra I, Lázaro E, Martinez O, López-Paz J, García M, Pérez M, Al-Rashaida M, Rodríguez Bermejo AA, Ovalle P, Perez-Nuñez P, Blanco-Lago R, Nevado J. Coping with Wolf-Hirschhorn syndrome: quality of life and psychosocial features of family carers. Orphanet Journal of Rare Diseases 2020; 15. |
|  |  | Rodríguez Bermejo AA, Martinez O, Amayra I, López-Paz J, Al-Rashaida M, Lázaro E, Caballero P, Pérez M, Berrocoso S, García M, Luna P, Perez-Nuñez P, Passi N. Diseases Costs and Impact of the Caring Role on Informal Carers of Children with Neuromuscular Disease. International Journal of Environmental Research and Public Health 2021; 18:2991. |
| China | 2 | Qi X, Xu J, Shan L, Li Y, Cui Y, Liu H, Wang K, Gao L, Kang Z, Wu Q. Economic burden and health related quality of life of ultra-rare Gaucher disease in China. Orphanet Journal of Rare Diseases 2021; 16. |
|  |  | Xu J, Bao H, Qi X, Wang J, Yin H, Shang C, Tan R, Wu Q, Huang W. Family caregivers of rare disease: A survey on health‐related quality of life in family caregivers for Gaucher disease patients in China. Molecular Genetics & Genomic Medicine 2021; 9. |
| Korea | 1 | Kim KR, Lee E, Namkoong K, Lee Y, Kim H. Caregiver's Burden and Quality of Life in Mitochondrial Disease. Pediatric neurology 2010; 42:271-6. |
| Canada | 3 | McMillan HJ, Gerber B, Cowling T, Khuu W, Mayer M, Wu JW, Maturi B, Klein-Panneton K, Cabalteja C, Lochmuller H. Burden of Spinal Muscular Atrophy (SMA) on Patients and Caregivers in Canada. Journal of Neuromuscular Diseases 2021; 8:1-16. |
|  |  | Currie G, Szabo J. “It is like a jungle gym, and everything is under construction”: The parent's perspective of caring for a child with a rare disease. Child: Care, Health and Development 2018; 45. |
|  |  | Baumbusch J, Mayer S, Sloan-Yip I. Alone in a Crowd? Parents of Children with Rare Diseases’ Experiences of Navigating the Healthcare System. Journal of Genetic Counseling 2018; 28. |
| Croatia | 1 | Čagalj D, Buljevac M, Leutar Z. Being a Mother of a Child with Prader-Willi Syndrome: Experiences of Accessing and Using Formal Support in Croatia. Scandinavian Journal of Disability Research 2018; 20:228-37. |
